# The Human Operculo-Insular Cortex Is Pain-Preferentially but Not Pain-Exclusively Activated by Trigeminal and Olfactory Stimuli

**DOI:** 10.1371/journal.pone.0034798

**Published:** 2012-04-05

**Authors:** Jörn Lötsch, Carmen Walter, Lisa Felden, Ulrike Nöth, Ralf Deichmann, Bruno G. Oertel

**Affiliations:** 1 pharmazentrum frankfurt/ZAFES, Institute of Clinical Pharmacology, Goethe - University, Frankfurt am Main, Germany; 2 Brain Imaging Center, Goethe - University, Frankfurt am Main, Germany; University Of Cambridge, United Kingdom

## Abstract

Increasing evidence about the central nervous representation of pain in the brain suggests that the operculo-insular cortex is a crucial part of the pain matrix. The pain-specificity of a brain region may be tested by administering nociceptive stimuli while controlling for unspecific activations by administering non-nociceptive stimuli. We applied this paradigm to nasal chemosensation, delivering trigeminal or olfactory stimuli, to verify the pain-specificity of the operculo-insular cortex. In detail, brain activations due to intranasal stimulation induced by non-nociceptive olfactory stimuli of hydrogen sulfide (5 ppm) or vanillin (0.8 ppm) were used to mask brain activations due to somatosensory, clearly nociceptive trigeminal stimulations with gaseous carbon dioxide (75% v/v). Functional magnetic resonance (fMRI) images were recorded from 12 healthy volunteers in a 3T head scanner during stimulus administration using an event-related design. We found that significantly more activations following nociceptive than non-nociceptive stimuli were localized bilaterally in two restricted clusters in the brain containing the primary and secondary somatosensory areas and the insular cortices consistent with the operculo-insular cortex. However, these activations completely disappeared when eliminating activations associated with the administration of olfactory stimuli, which were small but measurable. While the present experiments verify that the operculo-insular cortex plays a role in the processing of nociceptive input, they also show that it is not a pain-exclusive brain region and allow, in the experimental context, for the interpretation that the operculo-insular cortex splay a major role in the detection of and responding to salient events, whether or not these events are nociceptive or painful.

## Introduction

Studying or modulating pain in the brain requires the specific knowledge of the regions in which pain rather than just sensory input is processed. So far, several studies, often employing functional magnetic resonance imaging (fMRI) techniques, have shown that the administration of pain is associated with activations in a complex network of brain structures [1], [2] referred to as the “pain matrix”:. It includes, among other regions, the thalamus, the insular, anterior cingulate, primary and secondary somatosensory, premotor and supplementary motor cortices [3], [4], [5], [6]. However, since several studies have shown that most parts of the “pain matrix” are also activated by non-painful stimuli [7], [8], [9], the representation of pain in the brain has again become a subject of scientific discussion. To approach the specific representation of pain in the brain, non-painful somatosensory, auditory or visual stimuli were used to control for confounding non-pain specific activations [7], [8], [9]. Employing this paradigm, assessments using PET, evoked potentials or MRI techniques pointed at the operculo-insular cortex as major pain-specific part of the pain matrix [10], [11], [12], [13].

The pain-specificity of the operculo-insular cortex can thus be verified by administering nociceptive and non-nociceptive stimuli. This can be achieved by using somatosensory stimuli around the pain threshold as previously shown [12], or by using non-somatosensory stimuli, which is an accepted paradigm in pharmacological fMRI studies [14] to control for non-pain specific changes in brain activations. In the latter case, the experimental techniques of stimulus administration should preferably be identical to minimize confounding factors. This can be achieved by exploiting nasal chemosensation [15], [16], which comprises the perception of both trigeminal and olfactory stimuli [17] that can be similarly administered [18]. While similarities and differences between brain activations following trigeminal or olfactory stimuli have been repeatedly addressed before [19], [20], [21], [22], the focus in the present fMRI assessments was on pain-specificity of the brain activations while olfactory stimuli were only applied to control for confounding non-pain specific activations.

This approach served to investigate whether the operculo-insular cortex can be regarded as a specific brain area processing nociceptive input, where “specific” was used as “belonging or relating uniquely to a particular subject” (http://oxforddictionaries.com/, accessed on February 6, 2012), which in the present case was pain. This implied pain-exclusive activations, i.e., only activated by nociceptive stimuli whereas an alternative was “pain predominance”, i.e., “present as the strongest or main element”, however, not completely excluding activations by non-painful stimuli.

## Methods

### Subjects and study design

The study followed the Declaration of Helsinki on Biomedical Research Involving Human Subjects and was approved by the Ethics Committee of the Medical Faculty of the Goethe - University, Frankfurt am Main, Germany. All subjects had given informed written consent. Twelve healthy, right-handed subjects (eight men, four women), aged 24–34.8 years (mean ± standard deviation: 28.3±3 years) with normal body mass index (23.7±1.4 kg/m^2^) were enrolled. The subjects' health was verified by medical interview and a short examination. An established olfactory test (“Sniffin' Sticks” test; Burghart Messtechnik GmbH, Wedel, Germany [23]) was performed to verify that all subjects had a normal sense of smell. Medications, except oral contraceptives, were prohibited for one week, and alcohol for 24 h, before the experiments.

### Stimulation procedures

All stimuli were applied to the subject's right nostril by means of an olfactometer (OM/2, Burghart Messtechnik GmbH, Wedel, Germany). The olfactometer allowed for precise control of the stimulus parameters, such as stimulant concentration, duration, steepness of onset (<50 ms) and airflow (8 l/min). All stimulants were embedded in a constantly flowing airstream (8 l/min) at controlled temperature (36.5°C) and humidity (80% relative humidity). Short gaseous CO_2_ stimuli (500 ms, 75% v/v) were used as nociceptive stimulant. Applied to the nasal mucosa, where CO_2_ is converted into bicarbonate and protons by the enzyme carboanhydrase [24], it evokes a short stinging pain sensation [25] due to excitation of trigeminal nociceptors [26], [27] via activation of TRPV1 [28], TRPA1 [29] and possibly further targets (e.g., acid sensitizing ion channels and proton receptors). The CO_2_ stimuli activate predominantly trigeminal A_δ_-fibres, with co-activation of C-fibres [30]. A CO_2_ concentration of 75% v/v was chosen to obtain reliably sensations well above the pain threshold which has been shown to be exceeded already at concentrations below 50% v/v CO_2_ [25].

For the delivery of non-nociceptive olfactory stimuli, gaseous H_2_S and vanillin were used at concentrations of 5 ppm and 0.8 ppm, respectively, which produce a clear smell that cannot be perceived by anosmic subjects [31], [32]. The occurrence of trigeminal co-stimulation induced by these stimuli can be excluded for the following reason: it has been shown that unilateral application of these stimuli does not allow to identify the stimulated side, which would be possible if a trigeminal component was present [18].

The subjects received all three types of stimuli (CO_2_, H_2_S, and vanillin) successively in a pseudo-randomized order, ensuring that each stimulus type occurred the same number of times (n = 25). Although the olfactometer has been shown to provide a method of stimulus administration that does not concomitantly excite mechanical or thermal receptors in the nasal cavity [27], to remove the impact of any residual stimulus on the data, “blank” stimuli were included, during which no stimulant was added to the air flow, keeping all other experimental parameters constant. Blank stimuli (n = 30) were randomly included between the chemosensory stimuli. To keep habituation and adaptation to the chemosomatosensory stimuli low [33], long randomly-spaced intervals of 13.5–26.7 s (mean ± standard deviation: 18.9±3.5 s) were used. After each olfactory or pain stimulus, subjects rated its intensity with respect to pain, smell or pleasantness by means of visual analog scales (VAS), displayed randomly within 3.4–6.6 s (mean ± standard deviation: 4.9±0.8 s) after stimulus presentation and ranging from “no pain” to “pain experienced at maximum” (which implies that somatosensory stimuli below the pain threshold would have been rated as “zero”), “no odour” to “intensive odour” or “unpleasant” to “very pleasant”. It should be noted that subjects were not asked for any rating after blank stimuli. The reason is that subjects were asked to interrupt the experiments by pressing an alarm button if no stimulus was perceived before a query, thus avoiding the risk of acquiring useless data due to equipment malfunction (such as a lost connection or a poor localisation of the Teflon tube in the nasal cavity). This was incompatible with querying the blanks. To keep the experiments short, one rating of a stimulus was requested at a time; equally often for pain, odour or pleasantness and at random order. This allowed data acquisition in a single fMRI session taking less than 1 h. It should be noted that the unimodal approach based on chemosensory stimuli as employed in this study differs from a recent comparable multimodal approach to pain-specific brain areas using somatosensory, visual and acoustic stimuli [34]. In the latter study, the somatosensory stimuli had been divided into nociceptive stimuli consisting of pulses of radiant heat generated by an yttrium laser with duration of 5 ms and strength of 3 J, and non-nociceptive stimuli consisting of constant current square-wave electrical pulses of 1 ms duration and 6 mA strengths.

### Functional magnetic resonance tomography

#### Image recording

The blood oxygenation level dependent (BOLD) response to each stimulus was recorded at a field strength of 3 T on a dedicated head scanner (Siemens Magnetom Allegra, Siemens Medical Solutions, Erlangen, Germany) equipped with a 4-channel transmit-receive head coil. To reduce motion artefacts, the subject's head was immobilized using foam pads. For acquisition of fMRI data, a T_2_^*^-weighted gradient echo (GE) echo planar imaging (EPI) sequence with the following parameters was used: TR = 2048 ms, TE = 30 ms, flip angle = 90°, echo spacing = 420 µs, matrix size = 64×64, field of view = 192×192 mm^2^, and in-plane resolution = 3×3 mm^2^. A total of 750 volumes was acquired, each of which comprised 32 slices with 3 mm thickness and an inter-slice gap of 1 mm, acquired in descending order; the first five volumes were discarded to ensure steady state conditions for the fMRI evaluation. To improve the BOLD sensitivity in the amygdala and in the temporal lobes, slices were tilted by −30° (axial towards coronal orientation), positive phase encoding blips were chosen, and a z-shim gradient with a moment of 1 mT/m·ms was applied as suggested previously [35].

For subsequent off-line correction of distortions in the EPI images due to inhomogeneities of the static magnetic field B0 [36], [37], magnetic field mapping was performed based on GE imaging with identical geometric parameters and with two different TE values (4.89 and 7.35 ms) from which magnitude images and a phase difference map were calculated directly on the scanner. In addition, a T_1_-weighted anatomical data set with 1 mm isotropic resolution was acquired for each subject using a three-dimensional (3D) magnetization prepared rapid gradient echo (MP-RAGE) [38] sequence with the following parameters: TR = 2200 ms, TE = 3.93 ms, flip angle = 9°, TI = 900 ms, field of view = 256×256 mm^2^, one slab with 160 sagittal slices of 1 mm thickness, using parallel acquisition (GRAPPA [39]) with an acceleration factor of 2 in phase encoding direction.

#### Image preprocessing

Functional magnetic resonance brain image processing and statistical analyses were performed with the statistical parametric mapping software SPM8 (version for Linux 64 bit, Wellcome Department of Imaging Neuroscience, London, UK; http://www.fil.ion.ucl.ac.uk/spm [40], [41], running on Matlab version 2011a for 64-bit Linux, Mathworks, Natick, MA, USA). All volumes of the EPI-sequence were realigned to the first volume [40] and unwarped using a field map generated from the individual phase difference maps and magnitude images acquired with the field mapping sequence [36], [37]. The high-resolution T_1_-weighted anatomical data set was co-registered to the mean EPI data set (which was created during the realignment and unwarping process), segmented and normalized using 4^th^-degree B-spline interpolation to obtain image voxel sizes of 3×3×3 mm^3^. The resulting spatial normalization parameters were applied to the individual volumes of the EPI time series and subsequently smoothed with an isotropic 12 mm full-width-at-half-maximum Gaussian kernel [42].

#### Identification of stimulus associated activations

A general linear model [43] was used to partition the observed neurophysiological responses into components of interest, confounds and errors. An event-related analysis estimated the BOLD responses evoked by the CO_2_, H_2_S, vanillin or blank stimuli by modelling them as Heaviside functions, with stimulus durations as parameters, convolved with the canonical hemodynamic response function (HRF) as implemented in SPM8. The visual request for stimulus rating and the subsequent button-press, recorded by the “Presentation” software (Neurobehavioral Systems, Albany, USA), were modelled within the design matrix but omitted from second level analysis. This included unqueried button presses that accidentally occurred between ratings (median [interquartile range]: 3 [2.75–5] per subject). Furthermore, the six rotational and translational parameters from the rigid body transformation, obtained during image realignment, were modelled as covariates of no interest. The serial autocorrelation of the BOLD time series was modelled using a first-order autoregressive model. Low-frequency fluctuations of the MR signal were removed with a high-pass filter set to 128 Hz. Effects were tested with linear contrasts resulting in *t*-statistics for every voxel. The stimulus-associated activation patterns were obtained by generating contrast images for each subject and sensory stimulus class, versus the activations following blank stimuli (1 0 0 −1 for CO_2_, 0 1 0 −1 for H_2_S and 0 0 1 −1 for vanillin. The chosen order of the stimuli and the coding of blanks as −1 will be maintained for all contrast descriptions throughout this work.

### Statistical evaluation

It was tested if the CO_2_ stimuli were perceived as painful/not smelling, whereas the H_2_S and vanillin stimuli were perceived as smelling/not painful, using non-parametric analysis of variance on ranks or Wilcoxon tests (software: Stata/IC version 12 for Linux, StataCorp, College Station, TX, USA). The SPM8-based analysis of the fMRI data included the simple contrast maps derived from each participant in a random-effects second level analysis, which used a flexible factorial design and subsequent contrast analysis of “subject” and “stimulus” [44], although “subject” was omitted from the reports. The subsequent statistical analysis was based on the following considerations: An obvious approach to detecting pain-specific brain activations would be to exclusively mask the activations associated with the CO_2_ stimuli with those associated with the H_2_S or the vanillin stimuli. However, this procedure may yield the following problems: As the observation of brain activations in fMRI implies a statistical threshold, the HRF of activated and non-activated regions may differ from noise at p = 0.049 and p = 0.051, respectively. Thus, non-significant activations may be only slightly less pronounced than significant activations, providing a relatively weak basis for pain-specificity. Furthermore, the fact that an olfactory stimulus does not yield significant activation in a certain brain area can also result from lower cross-modality stimulus intensity without necessarily implying that the particular region will never be significantly activated by olfactory stimuli at higher intensities. Therefore, better evidence for a pain-specificity of a brain region was considered to be obtained when additionally requiring stronger activations following nociceptive than non-nociceptive stimuli. Thus, the analysis is based on the detection of regions that show (i) significantly higher activation following nociceptive than following non-nociceptive stimulation and (ii) no significant activation following non-nociceptive stimuli. Both conditions were required to consider a region as “pain specific”. This procedure should circumvent a major part of the methodological limitations discussed above. It should be noted that the procedure does not exclude but rather includes the requirement that the region should not be activated by non-nociceptive stimuli. That is, all regions with exclusive activations following nociceptive trigeminal stimuli are more activated by pain than by non-nociceptive stimuli since the latter provide an activation of zero at these regions, which is obviously smaller than activation above zero. Based on this reasoning, regions displaying stronger activations after trigeminal than after olfactory stimulations were identified by a conjunction of the contrasts for CO_2_>H_2_S and CO_2_>vanillin. Regions activated by pain but not smell could be approached by exclusively masking CO_2_-associated activations with the activations following olfactory stimulation. The latter coincided with activations following H_2_S stimulation that completely included regions activated following vanillin stimulation (no activated regions remained when masking the contrast 0 0 1 −1 exclusively with the contrast 0 1 0 −1). The corresponding psychophysical ratings were analysed using repeated measures analysis of variance on ranks and Wilcoxon tests. The statistical parametric maps (SPMt) resulting from the fMRI analyses were interpreted regarding the probabilistic behaviour of Gaussian random fields [45]. Clusters >3 voxels [46] that were significant at various successively applied Family Wise Error (FWE [47]) corrected α levels (p<0.05, p<0.01, p<0.001) are reported as Montreal Neurological Institute (MNI) coordinates (mm). Mean percent signal changes associated with stimuli were calculated for 5 mm spherical search volumes around selected peak coordinates (rfxplot toolbox [48]). The localization of brain activations was aided by the automatic anatomic labelling toolbox for SPM8 [49] and the Anatomy toolbox (version 1.8 [50], [51], [52]).

## Results

**Figure 1:**
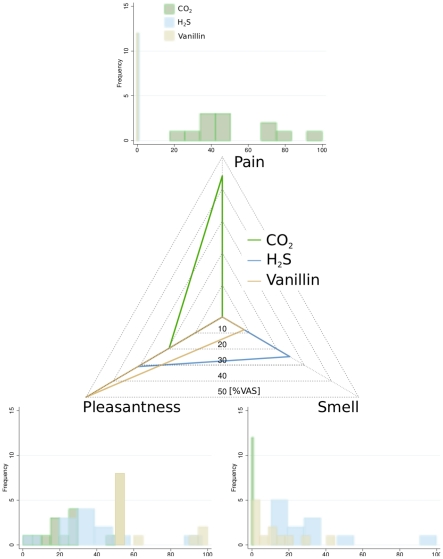
Estimates of the CO_2_, H_2_S and vanillin stimuli with respect to pain, smell or pleasantness, rated by means of a visual analog scale displayed at 3.4–6.6 s after stimulus presentation and ranging from “no pain” to “pain experienced at maximum” or “no odour” to “intensive odour” or “unpleasant” to “very pleasant”. CO_2_ was always painful and never smelled. H_2_S or vanillin stimuli were never painful but smelled.

The subjects perceived the CO_2_ stimuli as painful (median [interquartile range]: 44 [37.8, 73.4] % VAS pain) but not smelling (0 [0.0, 0.0] % VAS smell) and the H_2_S or vanillin stimuli as smelling but never painful (0 [0.0, 0.0] % VAS pain) (Figure 1). H_2_S smelled more than vanillin (24.8 [15.7, 33.2] versus 8.0 [0.0, 17.5] % VAS smell; Wilcoxon test: p = 0.012). Vanillin was most pleasant (50.0 [50.0, 70.0] % VAS pleasantness), H_2_S slightly and CO_2_ very unpleasant (31.0 [28.5, 45.0] and 19.5 [15.0, 27.0] % VAS pleasantness; repeated measures analysis of variance on ranks: p<0.001, all pairwise comparisons: p<0.05).

**Figure 2:**
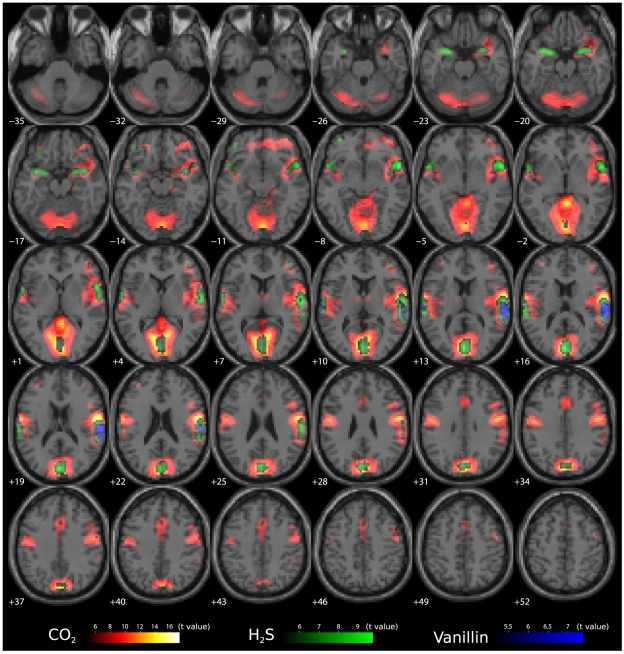
Activations following nociceptive and non-nociceptive stimuli. The trigeminal CO_2_ stimuli were associated with extended brain activations (contrast 1 0 0 −1 denoting CO2, H2S, vanillin and blank stimuli associated responses respectively). The significance at voxel level is colour-coded from dark red, low, to yellow, high, with increasing t values. Significant activations associated with the olfactory stimuli (contrasts 0 1 0 −1 and 0 0 1 −1) were less pronounced and less extended across the brain (colour depth of the displayed voxels reflects the respective t value of the voxel from dark green, low, to light green, high, for the H_2_S stimuli associated activations, and dark blue to light blue for the vanillin associated activations). The vanillin stimuli associated activations (blue) were completely within regions covered by the H_2_S associated activations (green) and both were almost within CO_2_ stimuli associated activations (red/yellow). Therefore, the pain associated activations were significantly stronger than the olfactory stimuli associated ones only in restricted regions, including the right insular cortex and bilaterally the rolandic operculum containing S2. Voxels are shown at a threshold of p<0.01 (FWE-corrected; t>5.091).

**Figure 3:**
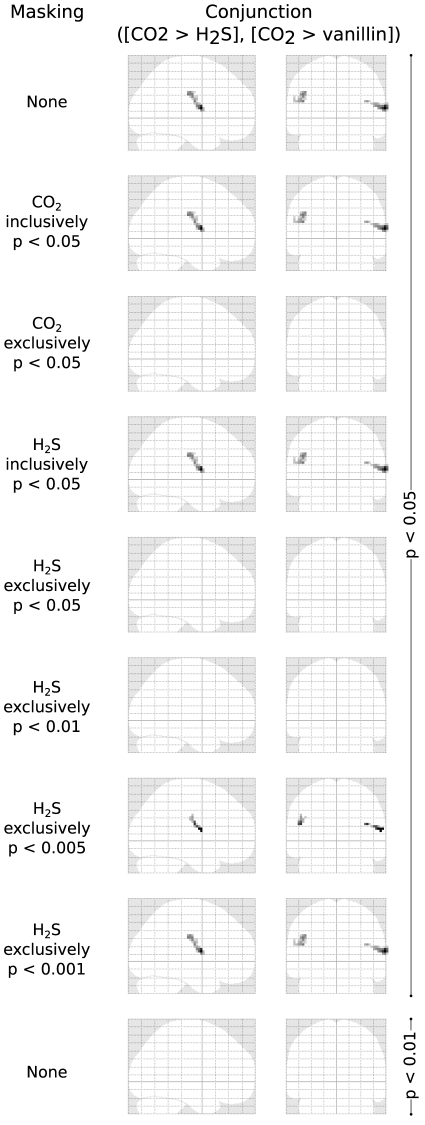
Pain-stimulus associated brain activations where the contrast pain > smell was significant (conjunction of second-level t-contrasts 1 −1 0 and 1 0 −1 denoting CO_2_, H_2_S and vanillin stimuli associated responses respectively). Statistically significantly activated voxels (p<0.05 FWE-corrected; t>5.14) are presented reflecting a 12-subject group analysis. Al the bottom line, the significance level was increased to FWE p<0.01. Effects of different masking of the pain > smell activations with different activations associated with pain (CO_2_) or smell stimuli (the respective masking is indicated at the left side of the glass brains) at various FWE corrected significance levels of the mask, and the effect of increasing the FWE corrected significance level of the pain > smell activation to p<0.01. At the top of the figure, the pain > smell contrast is unmasked. In the glass brains following below, this contrast is masked inclusively or exclusively with regions significantly activated following CO_2_ or H_2_S stimuli. As the regions showing activations associated with H_2_S stimuli completely covered those showing activations associated with vanillin stimuli, the first were taken as activations associated with smell. As expected, the activations pain > smell were within activations following CO_2_ stimulation (inclusive masked) and not outside them and therefore disappeared when exclusively masked with the CO_2_ associated activations. Unexpectedly, the activations pain > smell were also within activations following olfactory (H_2_S) stimulation (inclusively masked) and disappeared when exclusively masked with H_2_S associated activations, i.e., they were not outside the regions significantly activated by olfactory stimuli.

Localisations of brain activations associated with the nociceptive trigeminal stimuli or olfactory stimuli are shown in Figure 2. Although the results suggest that pain apparently evoked much more extended activations than the olfactory stimuli, brain regions showing significantly (FWE corrected p<0.05) stronger activations associated with CO_2_ stimuli than with non-nociceptive olfactory stimuli were found only within restricted brain areas. Specifically, two clusters were identified comprising 40 and 33 voxels (Table 1 and Figure 3) and containing the primary (areas 3a and 3b [53]) and secondary somatosensory areas (assigned to OP3 and OP4 [54]) and the insular cortex (right lobe) . The global maximum of activations was at the right side resembling the previously shown right-hemisphere preponderance of the activations associated with the CO_2_ stimuli [16], [55].

**Table 1:** Clusters of brain regions, which were activated more following pain than following non-nociceptive stimuli (Conjunction of CO_2_>H_2_S and CO_2_>Vanillin).

	Number of voxel in cluster	Peak coordinates			t value of peak coordinates
Brain regions within the cluster		x	y	z	
Right primary somatosensory cortex*	40	66	−1	13	7.38
Right secondary somatosensory/insular cortex*		42	−10	22	6.59
Left primary somatosensory cortex	33	−45	−19	31	6.74
		−57	−7	25	6.38
Left secondary somatosensory cortex		−45	−10	22	6.72

The table contains the anatomic location of the voxels with highest voxel level t in the respective region of a 12-subject group analysis. Voxels are given at a threshold of p<0.05 family wise error (FWE) corrected. Coordinates are reported in the MNI space [mm].

* contralateral to the stimulus application side.

**Figure 4:**
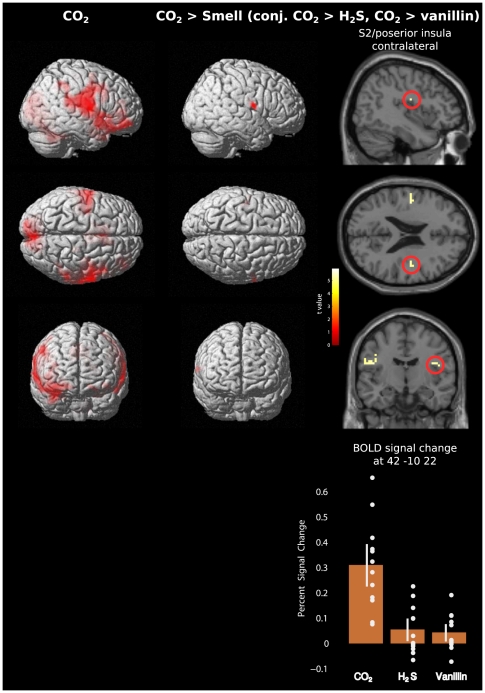
Brain activations observed following the intranasal administration of 500 ms pulses of gaseous CO_2_ at 75% v/V, which was clearly above pain threshold. **Left column:** Pain-stimulus associated activations. **Middle and right column:** Pain-stimulus associated brain activations where the contrast pain > smell was significant (conjunction of second-level t-contrasts 1 −1 0 and 1 0 −1 denoting CO_2_, H_2_S and vanillin stimuli associated responses respectively). The left Rolandic operculum and insular cortex contralateral to the stimulation displayed these pain predominant activations although activations were observed bilaterally. Statistically significantly activated voxels (p<0.05 FWE-corrected; t>5.14) are presented overlaid (red) on 3D surface renderings of a standard MNI brain (Panel A) and as coloured overlay on the horizontal and sagittal plane of a structural standard T1-weighted MRI template (left). In the right parts of the figure, the colour depth of the displayed voxels reflects the respective t value of the voxel. Furthermore, stimulus related brain activations corresponding to the different stimuli are reported as mean percent signal change in a 5 mm spherical search volume around a selected FWE-corrected peak coordinate (MNI 42 −10 22; bottom). Single subject activations are depicted as dots and the 95% confidence interval as white bars. Results reflect a 12-subject group analysis.

The two clusters expectedly persisted or disappeared when inclusively or exclusively, respectively, masked at FWE corrected p<0.05 with areas activated by nociceptive stimuli (Figure 3). However, they also persisted or disappeared when inclusively or exclusively, respectively, masked at FWE corrected p<0.05 with areas activated by H_2_S stimuli. A closer look into the activations in these regions showed that the 95% confidence intervals of the BOLD signal changes associated with H_2_S stimuli were above zero (Figure 4). This indicates that H_2_S stimulation produced activations in these regions, although significantly smaller than activations following nociceptive stimulation. When stepwise increasing the significance level of the H_2_S related exclusive masking to 0.001, activations associated with nociceptive stimuli reappeared (Figure 3). However, raising the significance level of the conjunction of contrasts for CO_2_>H_2_S and CO_2_>vanillin to FWE corrected p<0.01 had the consequence that activations following nociceptive stimulations were not identifiable anymore.

## Discussion

The operculo-insular cortex region met the first condition of the present approach to its role in pain and displayed stronger activations associated with nociceptive than with non-nociceptive stimuli. This agrees with previous studies where this region was recognized as important for pain perception [10], [11], [12], [22] and verified the localisation of brain activations observed in an early magneto-encephalography experiment [56]. The operculo-insular cortex had emerged as pain-preferential when using visual stimuli as non-nociceptive comparators [7]. Similarly, in a multisensory approach comparable with the present one but using different stimuli (nociceptive laser heat, non-nociceptive electrical currents, visual and auditory stimuli [34]), brain activations following nociceptive stimuli were found to be largely explicable by a combination of neural activities elicited by all stimuli regardless of the sensory modality [34].

While in that study [34], separate somatosensory non-nociceptive and somatosensory nociceptive stimuli had been used, the information about the difference in brain activations around the pain threshold for the present CO_2_ stimuli was already available from a previous study [12]. From this it was known that the activations in the operculo-insular region following the trigeminal nociceptive CO_2_ stimuli reflected both, the somatosensory perception of the stimuli regardless of their actual painfulness and the explicit perception of pain. This is true for all nociceptive stimuli as prior to crossing the pain threshold, they are already somatosensory as soon as their intensity has crossed the perception threshold. A distinction of the associated brain activations has been made previously when activations associated with CO_2_ stimuli were separated into components due to somatosensory stimulus administration independent of pain, components due to stimulus intensity independent of pain and components due to pain [12]. As a result of that investigation, the insular cortex could be attributed to processing both somatosensory stimulus intensity and nociceptive input.

The important functional association of the operculo-insular cortex with pain is further supported by the reported possibility to evoke pain by direct stimulation of the operculo-insular [57] or insular cortex [58]. Further reports support the hypothesis that the posterior insula is a major region of processing nociceptive input [7], [13], [59], [60], [61], [62], [63], [64], [65], [66], [67] including the description of operculo-insular pain as a distinct central pain syndrome [68]. However, the view of the operculo-insular cortex as a major pain-specific part of the pain matrix is not shared by all researchers. Clinical observations showed that the ability to evaluate pain can be retained despite substantial insular damage and no evidence of detectible insular activity [69].

However, the present results support only a pain-predominance of the operculo-insular cortex while the activations were not pain-exclusive. That is, when additionally applying the second criterion, i.e., controlling for non-pain specific activations by eliminating all regions activated by the olfactory stimuli, a complete extinction of all brain activations resulted. This could be attributed to small activations in these regions, including in the operculo-insular cortex, that were associated with the olfactory stimuli (Figure 3 bottom). Pain-exclusivity, however, would have implied that the activations were only obtained with nociceptive stimuli.

The insular cortex is extensively connected to other brain regions such as the prefrontal cortex, cingulate cortex, amygdala, parahippocampal gyrus, and secondary somatosensory cortex [70], [71], [72] and regarded as a relay in a functional cortical network processing saliency, switching, attention and control that is not restricted to pain [73]. A comprehensive analysis of observations in patients with insular damage, who display gustatory, olfactory, auditory and somatosensory disturbances, came to the conclusion that the insula might be a multimodal area playing a role as a convergence zone implicated in the coordination between internal and external information through emotional subjective awareness [74]. As the posterior insula has also been suggested to play a role in the sensory discrimination of pain [62], [63], it is likely that it is already activated by potentially noxious but not yet painful stimuli which are evaluated with respect to their painfulness. This is not a pain exclusive task and non-nociceptive information seems to be processed to modulate varying levels of appreciation of the nociceptive stimulus [69]. The present experimental conditions facilitated this comparative judgment as the intranasal trigeminal nociceptive stimuli and olfactory non-nociceptive stimuli were applied in a context where each stimulus could be expected to be painful. In addition, as a rating task was associated with each stimulus, the experiment extended the stimulus-associated brain activations [75], facilitating their detection. This may also involve decision processes about pain in the operculo-insular cortex, a hypothesis not verified by the present data, although based on demonstrations that the perception of a stimulus as nociceptive involves decisions [76], [77], [78], [79].

Under the present experimental conditions the activations following the administration of the nociceptive stimuli might have reflected both, pain or just the somatosensory perception of the stimuli [12]. As despite this possible confounder no pain exclusive activations were found, the results suggest that there is no pain-specific brain region at all. This seems to be compatible with the recent proposal of the pain matrix as a salience detection system for the body [80]. This judgment of the “pain matrix” was based on findings that the brain representation of the pain intensity could be dissociated from the representation of the dichotomous perception of a stimulus as painful or not [12], on a strong contextual modulation of the responses in the “pain matrix” to noxious stimuli [81], [82], and from the present and previous [83] observations that non-nociceptive stimuli can elicit cortical responses with a spatial configuration overlapping with that of the “pain matrix”. The present findings emphasize a predominant role of the operculo-insular cortex within the “pain matrix” as a network involved in detecting salient sensory events facilitating the processing of behaviourally significant and potentially threatening sensory input [80]. Apart from other regions [84], [85], the insular cortex was reported to be a part of this pain-associated saliency network [86]. However, the present experiment targeted pain as a unique perception [87]. The context of randomized stimulation made each stimulus a potential pain stimulus, so the activations in the operculo-insular cortex following the olfactory stimuli might reflect the evaluation of their pain-related saliency. This limitation, having arisen during interpretation of the study results, can only be solved in a follow-up study where subjects are exposed only to olfactory stimuli without potential pain. However, when using only acoustic stimuli in a typical oddball paradigm [34], a network of brain regions activated by the rarer stimuli was identified that largely overlapped with the network of brain regions displaying the multimodal BOLD responses identified with multimodal sensory stimuli, which emphasizes the importance of salience for the present observations. Finally, it has to be acknowledged that brain regions here labeled as being “pain preferential” because they responded more to trigeminal stimulation could also correspond to brain regions involved in the detection of and responding to salient events, regardless of whether these events are nociceptive, and regardless of whether these events are painful.

While nociceptive and olfactory stimuli were clearly distinguished with respect to the perception of either pain or smell, the other factors defining the olfactory quality were not as clearly distinguished, e.g. the pleasantness, where CO_2_ and H_2_S were more alike and vanillin contrasted most from the other stimulants. There are further qualities of odor, represented for example in the orbitofrontal and piriform cortices [88], which were not considered in the present analysis. However, none of those other qualities of odor was pain, so neglecting these qualities in this study did not influence the contrast of pain associated activations with non-pain associated activations. Similarly, while interactions between olfactory and trigeminal stimulations have been known for several years [15], [89], the stimuli were administered at considerable intervals to limit this interaction.

The analysis supported observations that the nociceptive preference of brain activations is limited to restricted areas. In line with the previously reported possibility to evoke pain by directly stimulating this area [57], it further identifies the operculo-insular cortex as a major brain region processing nociceptive input. However, the present experiments also show that the operculo-insular cortex is not exclusively activated by nociceptive stimulation but also involved in the processing of non-nociceptive stimuli, which allows for the interpretation that these brain regions are involved in the detection of and responding to salient events, whether or not these events are nociceptive or painful. Therefore, the results seem to be in line with a recent proposal of the “pain matrix” as a salience network involved in the evaluation of potentially threatening sensory input [34], [80] while emphasizing the role of the operculo-insular cortex within this network.
